# Development and Validation of a Brain Aging Biomarker in Middle-Aged and Older Adults: Deep Learning Approach

**DOI:** 10.2196/73004

**Published:** 2025-08-01

**Authors:** Zihan Li, Jun Li, Jiahui Li, Mengying Wang, Andi Xu, Yushu Huang, Qi Yu, Lingzhi Zhang, Yingjun Li, Zilin Li, Xifeng Wu, Jiajun Bu, Wenyuan Li

**Affiliations:** 1 Center for Clinical Big Data and Analytics, The Second Affiliated Hospital and Department of Big Data in Health Science, School of Public Health Zhejiang University School of Medicine Zhejiang University Hangzhou China; 2 Department of Radiology, The First Affiliated Hospital, Zhengzhou University First Affiliated Hospital of Zhengzhou University Zhengzhou China; 3 Department of Epidemiology and Health Statistics, School of Public Health, Hangzhou Medical College Hangzhou Medical College Hangzhou China; 4 School of Mathematics and Statistics, Northeast Normal University Northeast Normal University Changchun China; 5 Zhejiang Provincial Key Laboratory of Intelligent Preventive Medicine Hangzhou China; 6 Zhejiang Key Laboratory of Accessible Perception and Intelligent Systems College of Computer Science and Technology Zhejiang University Hangzhou China

**Keywords:** brain aging, deep learning, magnetic resonance imaging, MRI, imaging biomarker

## Abstract

**Background:**

Precise assessment of brain aging is crucial for early detection of neurodegenerative disorders and aiding clinical practice. Existing magnetic resonance imaging (MRI)–based methods excel in this task, but they still have room for improvement in capturing local morphological variations across brain regions and preserving the inherent neurobiological topological structures.

**Objective:**

To develop and validate a deep learning framework incorporating both connectivity and complexity for accurate brain aging estimation, facilitating early identification of neurodegenerative diseases.

**Methods:**

We used 5889 T1-weighted MRI scans from the Alzheimer’s Disease Neuroimaging Initiative dataset. We proposed a novel brain vision graph neural network (BVGN), incorporating neurobiologically informed feature extraction modules and global association mechanisms to provide a sensitive deep learning–based imaging biomarker. Model performance was evaluated using mean absolute error (MAE) against benchmark models, while generalization capability was further validated on an external UK Biobank dataset. We calculated the brain age gap across distinct cognitive states and conducted multiple logistic regressions to compare its discriminative capacity against conventional cognitive-related variables in distinguishing cognitively normal (CN) and mild cognitive impairment (MCI) states. Longitudinal track, Cox regression, and Kaplan-Meier plots were used to investigate the longitudinal performance of the brain age gap.

**Results:**

The BVGN model achieved an MAE of 2.39 years, surpassing current state-of-the-art approaches while obtaining an interpretable saliency map and graph theory supported by medical evidence. Furthermore, its performance was validated on the UK Biobank cohort (N=34,352) with an MAE of 2.49 years. The brain age gap derived from BVGN exhibited significant difference across cognitive states (CN vs MCI vs Alzheimer disease; *P*<.001), and demonstrated the highest discriminative capacity between CN and MCI than general cognitive assessments, brain volume features, and apolipoprotein E4 carriage (area under the receiver operating characteristic curve [AUC] of 0.885 vs AUC ranging from 0.646 to 0.815). Brain age gap exhibited clinical feasibility combined with Functional Activities Questionnaire, with improved discriminative capacity in models achieving lower MAEs (AUC of 0.945 vs 0.923 and 0.911; AUC of 0.935 vs 0.900 and 0.881). An increasing brain age gap identified by BVGN may indicate underlying pathological changes in the CN to MCI progression, with each unit increase linked to a 55% (hazard ratio=1.55, 95% CI 1.13-2.13; *P*=.006) higher risk of cognitive decline in individuals who are CN and a 29% (hazard ratio=1.29, 95% CI 1.09-1.51; *P*=.002) increase in individuals with MCI.

**Conclusions:**

BVGN offers a precise framework for brain aging assessment, demonstrates strong generalization on an external large-scale dataset, and proposes novel interpretability strategies to elucidate multiregional cooperative aging patterns. The brain age gap derived from BVGN is validated as a sensitive biomarker for early identification of MCI and predicting cognitive decline, offering substantial potential for clinical applications.

## Introduction

### Background

As human life expectancy steadily rises, the global population aged ≥60 years is projected to nearly double by 2050, reaching an estimated 2.1 billion [1]. This demographic shift presents substantial burdens to society, including rising health care costs and caregiving demands [2]. In the area of aging, dementia accounts for 11.2% of disability years, surpassing that caused by strokes (9.5%), musculoskeletal disorders (8.9%), cardiovascular diseases (5%), and all types of cancer (2.4%) [3]. Scientific investigations have illuminated the association between an aging brain and neurodegenerative conditions, such as Alzheimer disease (AD), by revealing the molecular and cellular mechanisms, particularly mitochondrial impairment [4,5]. There is an urgent demand for accurate assessment of brain aging to enable early detection of age-related neurodegenerative diseases, with neuroimaging-based brain age estimation methods gaining popularity in recent years [6].

Given its exceptional soft tissue contrast, noninvasive nature, and multimodal capabilities, magnetic resonance imaging (MRI) is the preferred method for timely visualizing brain structure and subtle lesions [7,8]. Brain age gap estimation [9], a quantified MRI-based score, has been considered a reliable brain age scale [10,11], with numerous studies associating a positive brain age gap with brain abnormalities and mortality [12,13]. The typical approach in this field involves constructing a standardized model using structural MRI data from healthy individuals. This model is then used to evaluate neuroanatomical deviations from the norm in new participants, providing insight into underlying brain abnormalities [14]. Recent publications have demonstrated the efficacy of traditional methods for brain age gap estimation, such as support vector regression and relevance vector regression, with mean absolute errors (MAEs) ranging from 2.6 years to 7.7 years [15] and 3.7 years to 4.7 years [16], respectively. However, these approaches often rely on supervised learning, with inherent limitations related to manual labeling and model performance influenced by dataset bias [14].

In recent years, the emergence and widespread adoption of deep learning (DL) techniques, which require no manual annotation and excel in handling high-dimensional data [17], have propelled model precision to unprecedented heights, achieving MAEs of <3 years [18-20]. For instance, BrainAgeNeXt [21] achieved an MAE of 2.78 years. Convolutional neural networks (CNNs) are lauded for their adeptness in automatic feature extraction from MRI images but are limited by the gradient explosion or vanishing issue in deeper layers. Residual neural networks (ResNets) have made significant strides by introducing skip connections to mitigate these training challenges, albeit at the cost of increased computational complexity. Transformer-based models represent a paradigm shift with their self-attention mechanisms adept at capturing long-range dependencies, but they demand substantial data for optimal training. Ensemble DL approaches have garnered attention for their ability to synthesize the predictions of multiple models, thereby enhancing the robustness and accuracy of brain age estimation. This prediction strategy, while beneficial for improving generalization, incurs the surcharge of increased training complexity. However, most DL-based approaches regard brain MRI as a cube constructed of voxels and then transfer models that perform well in natural images or videos directly to this task. This approach neglects the unique biological characteristics of the brain, which distinguish it from natural images [22], particularly in morphology [23]. Moreover, the human connectome exhibits intricate higher-order connectivity, encompassing spatial, functional, and sex-specific distinctions [24] among different brain voxel regions. Common CNN architectures designed for natural images lack the capacity to modify their topologies [25]. Currently, there is a notable absence of a DL framework that simultaneously considers both brain morphology and voxel regions connectivity for assessing brain aging. In addition, there is insufficient evidence to compare and quantify the clinical application value of the brain age gap and the relationship between narrowing MAEs with improving clinical application performance.

### This Study

Here we proposed a novel graph-based DL framework, a brain vision graph neural network (BVGN), which uses deformable kernels to extract brain morphological features and incorporated a graph neural network (GNN) to characterize voxel regions connectivity. We pioneered the adaptation of vision GNN [26], a backbone widely researched in natural image processing, to the domain of brain aging by extending its framework to 3D MRI. The BVGN leveraged an individual’s standard T1-weighted MRI as input and generated the corresponding neuroimaging biomarker-brain age gap to evaluate brain aging. Subsequently, we constructed multiple logistics regression models, each incorporating different types of input variables, to classify data samples labeled as cognitively normal (CN) and with mild cognitive impairment (MCI). We aimed to compare and validate the effects of brain age gap against conventional variables, including demographic factors, brain volume features, apolipoprotein E4 (APOE4) carriage, and general cognitive scales, in distinguishing CN and MCI states. In addition, we assessed the combined utility of the brain age gap with the Functional Activities Questionnaire (FAQ) cognitive assessment and the impact of lower MAEs. Cox proportional hazards regressions were performed separately using data from participants classified as CN and those with MCI at baseline to evaluate the potential of brain age gap as a risk biomarker for cognitive decline over a 2-year follow-up period.

## Methods

### Dataset, Study Design, and Inclusion Criteria

Data used in the preparation of this paper were obtained from the database of Alzheimer’s Disease Neuroimaging Initiative (ADNI) studies [27-29]. The ADNI was launched in 2003 as a public-private partnership, led by principal investigator Michael W Weiner, MD. The primary goal of ADNI has been to test whether serial MRI, positron emission tomography, other biological markers, and clinical and neuropsychological assessment can be combined to measure the progression of MCI and early AD.

**Figure 1:**
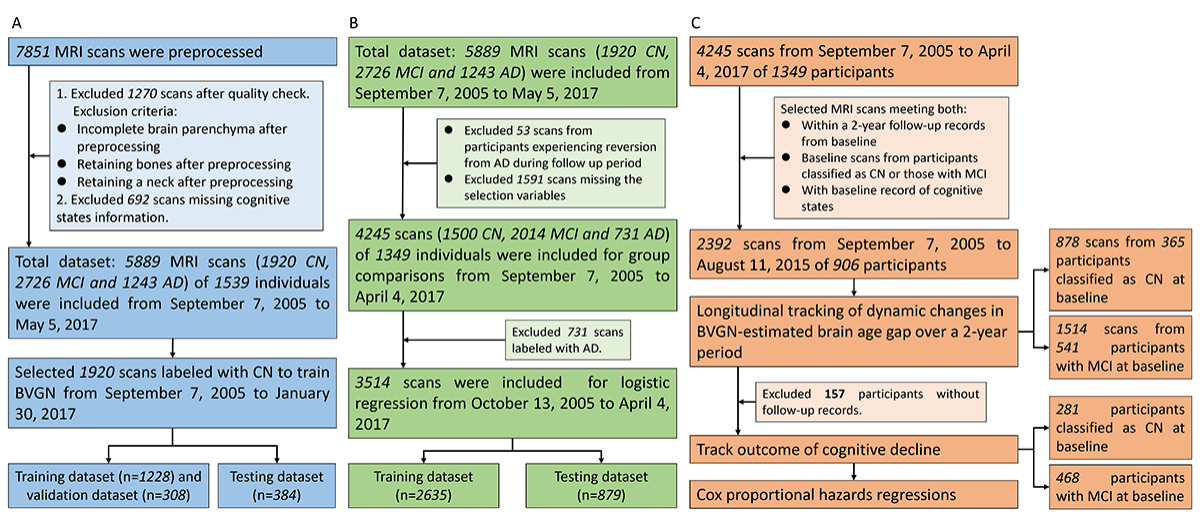
Flow diagram to show study design and inclusion criteria. (A) Brain vision graph neural network (BVGN) training and testing part; (B) cross-sectional validation part; (C) longitudinal validation part. AD: Alzheimer disease; CN: cognitively normal; MCI: mild cognitive impairment; MRI: magnetic resonance imaging.

This study gathered 7851 T1-weighted MRI scans from ADNI-1, ADNI-2, and ADNI-GO (Grand Opportunities) studies. The research design comprised 2 sequential phases: development and validation of the BVGN model for accurate brain age estimation, and clinical effect validation of the BVGN-estimated brain age gap metric by integrated cross-sectional and longitudinal analysis. For BVGN model development, we specifically chose 5889 scans from 1539 individuals that contained cognitive states labels and met both the ADNI MRI scanner protocols [30,31] and our strict training criteria (ie, presenting complete structural information after preprocessing). Data regarding the patient’s cognitive states were retrieved from the ADNI web portal [32]. Only 1920 MRI scans of participants classified as CN were used for training the BVGN model (Figure 1A). To ensure comprehensive model development and evaluation, we divided the dataset consisting of participants classified as CN into training set with validation set and test set in a ratio of 8:2. The training set and validation set were used for model training and hyperparameter setting, while the test set was used to validate the performance of the model. A subset of the UK Biobank (UKB) dataset [33] was used for external validation to evaluate model generalizability. We curated 34,352 MRI scans, partitioned into training set (n=21,985, 64%), validation set (n=5497, 16%), and test set (n=6870, 20%).

Given the progressive nature of dementia caused by neurodegenerative disorders, we excluded scans from participants who experienced reversion from AD during the follow-up period (Figure 1B). Following feature selection, 4245 MRI scans from 1349 participants were retained for cross-sectional analysis. All scans were used for intergroup comparisons, while only those from participants classified as CN and those with MCI were used for logistic regression analysis aiming at binary classification between CN and MCI. To validate the performance of logistic regression models, the dataset was split into training and test sets at an 8:2 ratio.

We combined various MRI scans from the same individual for longitudinal analysis (Figure 1C). Specifically, we used a dual-component longitudinal design to investigate the trajectories of BVGN-estimated brain age gap across cognitive progression groups and quantify cognitive decline risks through Cox proportional hazard models. For the brain age gap trajectory analysis, cognitive progression categories (CN-CN, CN-MCI or CN-AD, MCI-CN or MCI-MCI, and MCI-AD) were stratified based on recorded cognitive states from baseline to a 2-year follow-up. The inclusion criteria required participants to be classified as either CN or having MCI at the baseline, yielding 1514 scans from 541 participants with MCI and 878 scans from 365 participants classified as CN.

To evaluate time-dependent cognitive decline, we conducted Cox proportional hazards regression stratified by baseline groups. We excluded 157 participants without follow-up data and performed separate analyses for the baseline CN group (tracking MCI conversion) and MCI group (tracking AD conversion). The final analysis included 749 participants, comprising 281 (37.5%) individuals classified as CN and 468 (62.5%) individuals with MCI, with the time-to-event defined as the interval between baseline MRI and first observed cognitive transition.

### Quality Control and Data Preprocessing

For the ADNI dataset, each series within every examination was subjected to a rigorous quality control process at the Mayo Clinic. This involved 2 distinct levels of scrutiny—compliance with protocol-specific parameters and an assessment of the series-specific quality, such as the participant’s movement and the extent of anatomical coverage. The quality of the scans was evaluated by trained analysts who assigned a subjective grade—scores of 1 to 3 were considered satisfactory, while a score of 4 indicated a failure, rendering the scans unusable. While the UKB dataset was only used to test the model’s generalizability, all data also underwent standardized quality control procedures [34].

The acquisition protocols of different datasets required data preprocessing to ensure compatibility. The entire MRI preprocessing pipeline could be divided into 4 steps. First, the raw MRI data in neuroimaging informatics technology initiative format underwent minimum-maximum normalization. Second, nonbrain tissue, such as the skull and neck, was removed. In the third step, each MRI was registered from its native space to the standard Montreal Neurological Institute 152 1-mm^3^ template [35] chosen for registration in our study. Finally, central cropping was performed on the complete MRI volume, resulting in a final output size of 160×196×160 mm^3^. In addition, further preprocessing steps were conducted to exclude MRIs with incomplete brain structures, ensuring dataset quality. The preprocessing pipeline was implemented using SimpleITK [36] and FMRIB software library [37].

During the logistic and Cox regression modeling phase, *z* score normalization was applied to numerical variables, while categorical variables were transformed using one-hot encoding.

### BVGN Model Development

**Figure 2:**
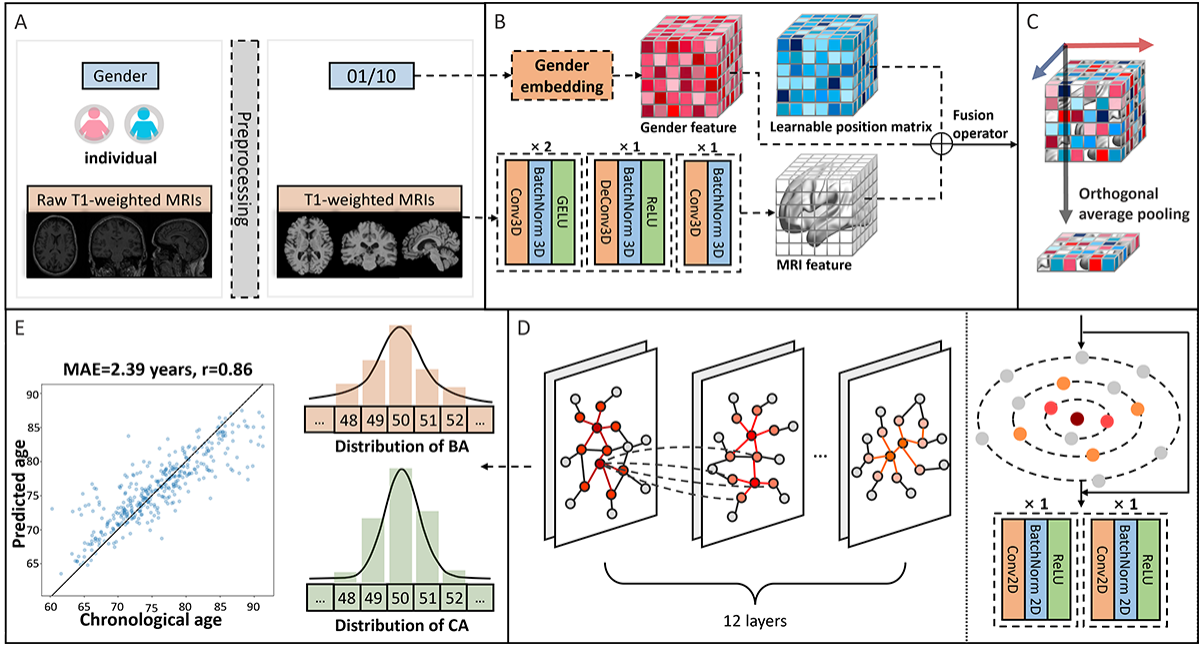
Overview of the brain vision graph neural network model. (A) A preprocessing pipeline was implemented, which involved converting gender into 1-hot encoding and transforming raw magnetic resonance imaging (MRI) data into brain tissue located in a standard space. (B) Discretization layer, the voxel space of brain is divided by the discretization layer, which uses a combination of multiple convolution modules and deformable convolution modules. Subsequently, positional features and gender features are integrated into cubes. (C) Orthogonal average pooling module (coronal axis, sagittal axis, transverse axis, the 3D feature cube is transformed into a 2D feature map). (D) Stacked graph neural network modules. (E) During training, the image on the right illustrates that the supervised signal of back-propagation is computed by evaluating the Kullback-Leibler divergence between estimated brain age and chronological age; whereas the image on the left depicts the model’s performance. BA: brain age; CA: chronological age.

Owing to the need for a large number of high-quality handcrafted features in traditional methods [14] and because inductive bias to brain structures is not effectively transferable from natural images when applying classical DL architectures, there are limitations in extracting features from irregular objects, such as cortical sulcus and gyrus, as well as parenchyma using grid-like convolution kernels that perform weighted summation of pixel values at the centroid and its neighbors [25,38,39]. Furthermore, CNN architectures lack the ability to modify topological relationships between voxel regions [22]. To address these issues, we proposed an end-to-end framework called BVGN (Figure 2), which used standardized MRIs to estimate brain aging of individuals. To be more specific, BVGN makes it easier to extract irregularly shaped structures in brain MRI by superimposing deformable convolution kernels. A graph CNN was used to model the relationship between different regions.

The BVGN framework consists of 3 components: discretization layer, stacked GNN layer, and aging distribution prediction header. Specifically, standardized MRIs were discretized into multiple cubes using nonoverlapping multiple deformable convolutional modules [38], where each cube’s feature vectors contained all information within the receptive field range of deformable convolutional modules. In addition, an individual’s sex and position information for each cube was incorporated into the feature map by passing through a projection module, modal-type embedding [40] and initializing a learnable parameter matrix [41]. An orthogonal average pooling module was implemented to project the 3D volumetric feature tensor along 3 mutually perpendicular anatomical axes (coronal, sagittal, and transverse). This dimensionality reduction operation transforms the 3D spatial representation into a 2D feature map while preserving critical spatial information across orthogonal planes. Detailed explanations of Figure 2 are provided in Multimedia Appendix 1.

The feature map was subsequently transformed into a graph using Euclidean distance and nearest neighbors as the basis, which was then input into a stacked GNN [26,42]. Importantly, the dynamic construction of the graph during forward processing relied on assessing similarity between feature maps at different depths. Ultimately, aging probability distributions [43,44] were obtained in the prediction header by integrating the feature map through bottlenecked convolutional kernels [45], based on predefined age ranges. Inspired by vision GNN [26], we implemented 2 distinct neural network architectures—the pyramid-shaped BVGN and the isotropic BVGN. The isotropic BVGN was used for interpretability analyses, while the pyramid-shaped BVGN was used to enhance overall performance. Both architectures were initiated with an identical discretization module featuring the same number of nodes. However, as the network deepened, the isotropic BVGN maintained a constant node count, ensuring uniform information flow akin to the transformer model’s approach. In contrast, the pyramid-shaped BVGN underwent a process of node consolidation, progressively reducing the number of nodes to form a pyramidal structure, echoing the hierarchical feature extraction of CNNs in computer vision.

### BVGN Model Training

The BVGN model was trained using multiple graphics processing units to ensure efficient computation. For optimization purposes, we implemented the stochastic gradient descent optimizer with an initial learning rate of 0.01, aiming to minimize the Kullback-Leibler divergence between the predicted age distribution and label age distribution. To prevent overfitting, an L2 weight decay coefficient value of 0.001 was applied during training sessions lasting up to 160 epochs. Furthermore, a batch size of 32 was adopted for improved performance, while progressively decreasing the learning rate by multiplying it with a factor of 0.7 after every consecutive span of 30 epochs.

### BVGN Model Performance and Generalizability Evaluation

**Figure 3:**
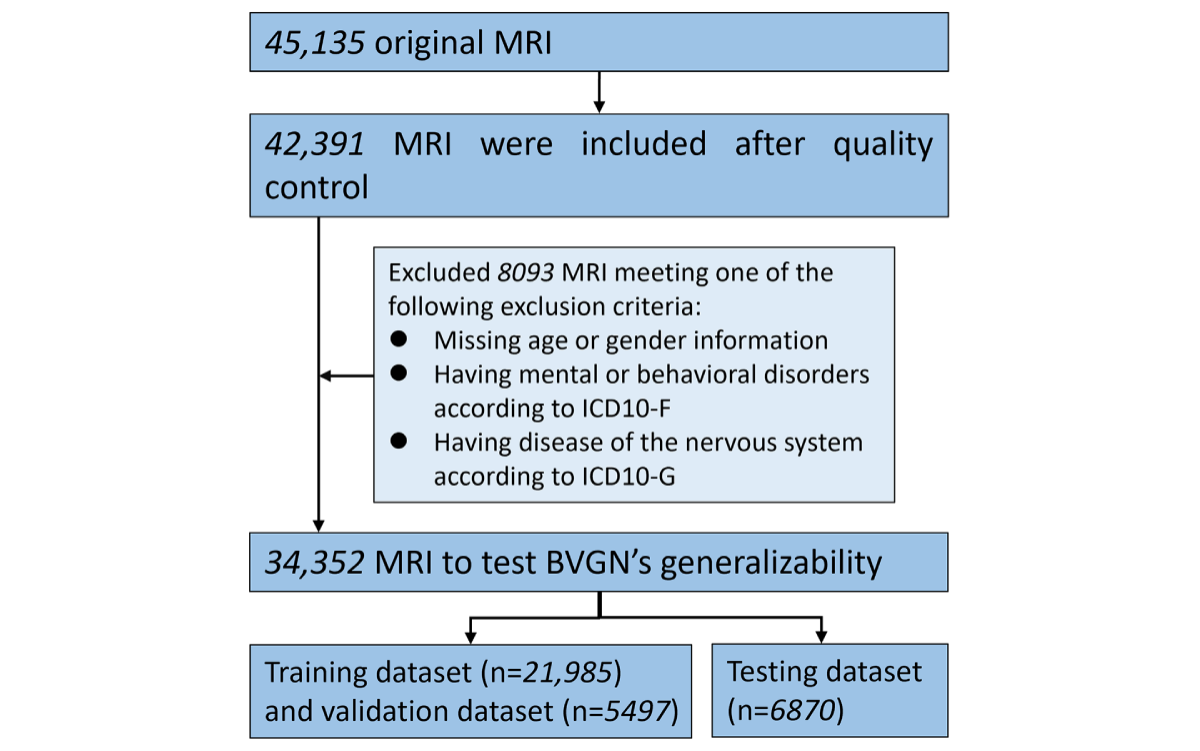
Flow diagram to show inclusion criteria for brain vision graph neural network’s (BVGN’s) generalizability. ICD: International Classification of Diseases; MRI: magnetic resonance imaging.

To objectively assess the performance of our framework, we used MAE and Pearson correlation as validation metrics to evaluate the efficacy of BVGN as a prediction model. Furthermore, we simultaneously trained and tested the simple fully convolutional network (SFCN) and ResNet models on the same dataset, ensuring a fair comparison. To validate the model’s generalizability, we retrained it using the UKB dataset, which is large scale and not restricted to disease-specific populations. The detailed inclusion process and demographic characteristics of the included UKB dataset are presented in Figure 3 and Table S1 in Multimedia Appendix 1.

### BVGN Model Interpretation

In contrast to models exclusively relying on CNNs, BVGN incorporates graph convolutional kernels to modify the relationships among local regions. This enables BVGN to offer interpretability not only through saliency maps but also by considering pertinent properties of the graph structure. More specifically, we first visualized a gradience-based saliency map [45] for participants classified as CN and those with MCI and AD. Subsequently, certified radiologists used the automated anatomical labelling atlas to ascertain the specific brain regions to which the saliency map corresponded. Furthermore, we conceptualized the brain as a graph-based complex system and characterized the interregional cooperative aging patterns by analyzing the connectivity of critical nodes across different hierarchical levels through data-driven visualization techniques.

### Cross-Sectional Analysis: Intergroup Comparisons and Logistic Regression

During cross-sectional analysis, each MRI scan was treated as an individual sample, and all statistical analysis was conducted accordingly. Numerical variables in demographics and selection features were presented as mean (SD), while categorical variables were presented as counts (n) and percentages (%). Statistical comparisons between training and testing sets were performed with 2-tailed significance. Specifically, the Student *t* tests and a 1-way ANOVA were used for numerical variables, and chi-square tests were used for categorical variables. A *P*<.05 was considered statistically significant.

For intergroup comparisons, we first characterized the distribution of the brain age gap using the median and IQRs across CN, MCI, and AD classifications. Subsequently, we used the Kruskal-Wallis test followed by 1-tailed Dunn test to evaluate potential significant differences in the brain age gap among these groups, with a significance level set at *P*<.05. Potential confounding effects were addressed through a multiple linear regression model incorporating age and gender as covariates.

We used multiple logistic regression models aiming at binary classification between CN and MCI samples. Our objective was to validate the diagnostic ability of the brain age gap compared to other conventional variables and to assess the potential clinical value of achieving lower MAEs in brain age gap estimation. Inspired by previous studies, we considered the brain age gap along with other relevant cognitive variables, including demographic characteristics, general cognitive assessments, brain volume features, and APOE4 carriage. Given the simplicity and high discriminative and predictive power of FAQ as reported by previous studies, we specifically investigated the impact of combining the brain age gap with FAQ to determine if their integration improves diagnostic classification.

Model performance was evaluated using metrics, including accuracy, precision, recall, *F*_1_-score, the receiver operating characteristic curves, and the area under the receiver operating characteristic curves (AUCs).

We initially established a benchmark model incorporating only demographic variables. Subsequently, brain age gap, various cognitive assessment scores, brain volume characteristics, and APOE4 carriage were added to the benchmark model to construct various logistic regression models. A comprehensive model integrating all the abovementioned variables was then developed, and their coefficients were compared. These analyses aimed to provide a holistic view in comparing the brain age gap with conventionally recognized cognition-related variables. Furthermore, we included both the FAQ and the brain age gap in the benchmark model to explore their combined utility. We also investigated the potential clinical value of lower MAEs by including diverse brain age gaps estimated from models with different precision, both in conjunction with the benchmark and comprehensive models, and compared their performances.

### Longitudinal Analysis: Brain Age Gap Trajectories and Risk Quantification

We conducted a longitudinal analysis to investigate changes in brain age gap across follow-up scans of the same participants and assess the risk of brain age gap for cognitive decline. We used BVGN, SFCN, and ResNet models to estimate brain age gap across the total 2392 scans and depicted how the estimated brain age gap changed over follow-up time in different progression groups.

After excluding participants without follow-up records, we performed Cox regression using the baseline brain age gap for both the CN and MCI groups. This allowed us to evaluate the association between brain age gap and cognitive decline, as well as to quantify its hazard ratio (HR). We initially estimated the HR of brain age gap using a univariate Cox proportional hazards model, followed by adjustment for demographic features to control for potential confounding bias. Subsequently, a multivariable Cox proportional hazards model that included all selected variables was used to assess the HR of the brain age gap from a holistic view. In addition, we plotted Kaplan-Meier survival curves stratified by the upper and lower 50% levels of brain age gap in both CN and MCI groups to visualize the performance of the brain age gap in predicting cognitive decline.

### Ethical Considerations

Data used in the preparation of this paper were obtained from the ADNI database. Consequently, the investigators within the ADNI contributed to the design and implementation of ADNI and provided data but did not participate in analysis or writing of this report. A complete listing of ADNI investigators can be found [46] on the internet.

Data collection and sharing for this project were funded by the ADNI (National Institutes of Health grant U01 AG024904) and DOD ADNI (Department of Defense grant W81XWH-12-2-0012).

The data used in the preparation of this paper were obtained from the UKB database. UKB has generic ethical approval from the North West Multi-centre Research Ethics Committee as a Research Tissue Bank (91486), and therefore researchers do not require separate ethical clearance to use the resource.

## Results

### Demographics and Selection Variable Characteristics

During the BVGN development process, the sample sizes for training, validating, and testing sets were 1228, 308, and 384, respectively. As shown in Table S2 in Multimedia Appendix 1, the mean ages for the training with validating sets and testing sets were 75.65 (SD 6.31) and 75.89 (SD 6.60) years, respectively. The percentages of male individuals in the training and validating set and testing set were 47.1% (724/1536) and 43.5% (167/384), respectively. No significant difference (*P*=.51) was observed.

Table 1 details the basic information of the dataset for intergroup comparison, which included 4245 MRI scans from 1349 participants. The discrepancy in participant count (1585 in Table 1) reflects instances where participants exhibited multiple cognitive manifestations during the follow-up period, leading to some being counted multiple times across groups. Specifically, the dataset comprised 1500 MRI scans from 499 participants classified as CN, 2014 scans from 709 participants with MCI, and 731 scans from 377 participants experiencing AD. The mean age was 75.39 years for participants classified as CN, 72.96 years for participants with MCI, and 74.76 years for participants with AD. The percentage of male participants was 46.1% (691/1500) for those classified as CN, 55.2% (1112/2014) for those with MCI, and 49.3% (360/731) for those with AD. Significant differences were observed between the CN, MCI, and AD groups for both age and gender characteristics.

**Table 1:** Demographic characteristics for the dataset of group comparisons.

			CN^a^		MCI^b^	AD^c^		*P* value	
MRI^d^ scans, n			1500		2014	731		—^e^	
Participants^f^, n			499		709	377		—	
Age (y), mean (SD)			75.39 (6.06)		72.96 (7.45)	74.76 (7.53)		<.001	
**Gender, n (%)**									<.001
	Male	691 (46.1)		1112 (55.2)		360 (49.3)			
	Female	809 (53.9)		902 (44.8)		371 (50.7)			

^a^CN: cognitively normal.

^b^MCI: mild cognitive impairment.

^c^AD: Alzheimer disease.

^d^MRI: magnetic resonance imaging.

^e^Not applicable.

^f^The total number of participants (n=1585) in Table 1 included instances where some participants had multiple cognitive manifestations during follow-up, leading to duplicates between groups. This total differed from the 1349 unique participants reported in Figure 1. Statistical analysis was performed by ANOVA test for age and chi-square test for gender. Two-tailed significance was used, with *P*<.05 indicating the presence of significance.

For the logistic regression modeling process, 3514 scans were included, with 2635 (74.99%) in the training set and 879 (25.01%) in the testing set. The characteristics of all variables used in the logistic regression are presented in Table 2. No significant differences were observed between training and testing sets for all the selected variables.

**Table 2:** Characteristics of selected variables in the training and testing datasets for logistic regression.

Variables		Total dataset (n=3514)	Training dataset (n=2635)	Testing dataset (n=879)	*P* value	
Education (y), mean (SD)		16.10 (2.81)	16.09 (2.82)	16.12 (2.79)	.77	
ADAS13^a^ (points), mean (SD)		12.50 (7.07)	12.56 (7.12)	12.32 (6.94)	.37	
MMSE^b^ (points), mean (SD)		28.22 (1.96)	28.23 (1.97)	28.19 (1.93)	.69	
RAVLT_Imm^c^ (points), mean (SD)		39.48 (11.99)	39.46 (12.04)	39.54 (11.85)	.86	
RAVLT_Learn^d^, (points), mean (SD)		4.89 (2.66)	4.87 (2.67)	4.97 (2.63)	.34	
RAVLT_Forget^e^ (points), mean (SD)		4.17 (2.69)	4.17 (2.67)	4.18 (2.75)	.96	
RAVLT_VF%^f^ (points), mean (SD)		48.97 (35.42)	49.09 (35.78)	48.60 (34.33)	.72	
TRABSCOR^g^ (points), mean (SD)		95.92 (56.17)	95.31 (55.33)	97.72 (58.60)	.27	
FAQ^h^ (points), mean (SD)		1.90 (3.50)	1.87 (3.45)	1.98 (3.65)	.41	
Ventricles (cm^3^), mean (SD)		36.39 (19.47)	36.48 (19.38)	36.10 (19.75)	.62	
Hippocampus (cm^3^), mean (SD)		6.99 (1.10)	6.98 (1.10)	7.02 (1.09)	.36	
Entorhinal (cm^3^), mean (SD)		3.62 (0.73)	3.60 (0.73)	3.65 (0.75)	.11	
Fusiform (cm^3^), mean (SD)		17.60 (2.59)	17.58 (2.56)	17.69 (2.69)	.28	
MidTemp (cm^3^), mean (SD)		19.70 (2.79)	19.71 (2.76)	19.68 (2.88)	.77	
ICV^i^ (cm^3^), mean (SD)		1524.19 (158.67)	1523.49 (157.98)	1526.28 (160.80)	.65	
BVGN_BAG^j^		3.74 (5.22)	3.79 (5.39)	3.58 (4.68)	.30	
SFCN_BAG^k^		3.71 (5.10)	3.73 (5.24)	3.66 (4.67)	.70	
ResNer_BAG^l^		4.24 (5.23)	4.30 (5.37)	4.08 (4.76)	.30	
**Gender, n (%)**						.51
	Male	1803 (51.3)	1343 (51.0)	460 (52.3)		
	Female	1711 (48.7)	1292 (49.0)	419 (47.7)		
**Marriage, n (%)**						.71
	Married	2628 (74.8)	1980 (75.1)	648 (73.7)		
	Widowed	400 (11.4)	289 (11.0)	111 (12.6)		
	Divorced	338 (9.6)	256 (9.7)	82 (9.3)		
	Never married	128 (3.6)	96 (3.6)	32 (3.6)		
	Unknown	20 (0.6)	14 (0.5)	6 (0.7)		
**APOE4_ε4^m^, n (%)**						.28
	0	2100 (59.8)	1594 (60.5)	506 (57.6)		
	1	1181 (33.6)	867 (32.9)	314 (35.7)		
	2	233 (6.6)	174 (6.6)	59 (6.7)		
**Cognitive states, n (%)**						.99
	CN^n^	1500 (42.7)	1125 (42.7)	375 (42.7)		
	MCI^o^	2014 (57.3)	1510 (57.3)	504 (57.3)		

^a^ADAS13: Alzheimer’s Disease Assessment—Cognitive Subscale 13-item version.

^b^MMSE: Mini-Mental State Examination.

^c^RAVLT_Imm: Rey Auditory Verbal Learning Test_immediate.

^d^RAVLT_Learn: Rey Auditory Verbal Learning Test_learning.

^e^RAVLT_Forget: Rey Auditory Verbal Learning Test_forgetting.

^f^RAVLT_VF%: Rey Auditory Verbal Learning Test_percentage_forgetting.

^g^TRABSCOR: trail making test part B, time (to complete in Neuropsychological Battery assessment).

^h^FAQ: Functional Activities Questionnaire.

^i^ICV: intracranial volume.

^j^BVGN_BAG: brain age gap estimated by the brain vision graph neural network model.

^k^SFCN_BAG: brain age gap estimated by the simple fully convolutional network model.

^l^ResNet_BAG: brain age gap estimated by the residual neural network model.

^m^APOE4_ε4: number of apolipoprotein E ε4 alleles.

^n^CN: cognitively normal.

^o^MCI: mild cognitive impairment.

### Performance and Generalizability of BVGN

We have simultaneously trained various strong baseline models alongside our proposed model using the same training set and explored their performance on the same test set. The results are shown in Table 3.

**Table 3:** Performance of benchmark models and the brain vision graph neural network (BVGN) model.

Models	Performance	
	MAE^a^ (y)	Pearson correlation coefficient (*r*)
3D ResNet15^b^	3.03	0.76
SFCN^c^	2.41	0.87
Isotropic BVGN	3.08	0.79
Pyramid BVGN	2.39	0.86
Pyramid BVGN (external validation)	2.49	0.92

^a^MAE: mean absolute error.

^b^ResNet15: residual neural network with 15 layers.

^c^SFCN: simple fully convolutional network.

Our isotropic BVGN attained an MAE of 3.08 years, which was slightly worse than the currently optimal model, SFCN, specifically designed for the brain age gap estimation (with an MAE of 2.41 years). After applying deformable convolutions, our pyramid BVGN achieved the best performance among all the tested models with an MAE of 2.39 years and a Pearson correlation coefficient of 0.86 between the predicted and chronological age.

The BVGN model, when retrained on the UKB dataset, exhibited robust performance with an MAE of 2.49 years and a Pearson correlation coefficient of 0.92 (Figure S1 in Multimedia Appendix 1), underscoring its strong generalization capacity.

### Interpretability Analysis of BVGN

**Figure 4:**
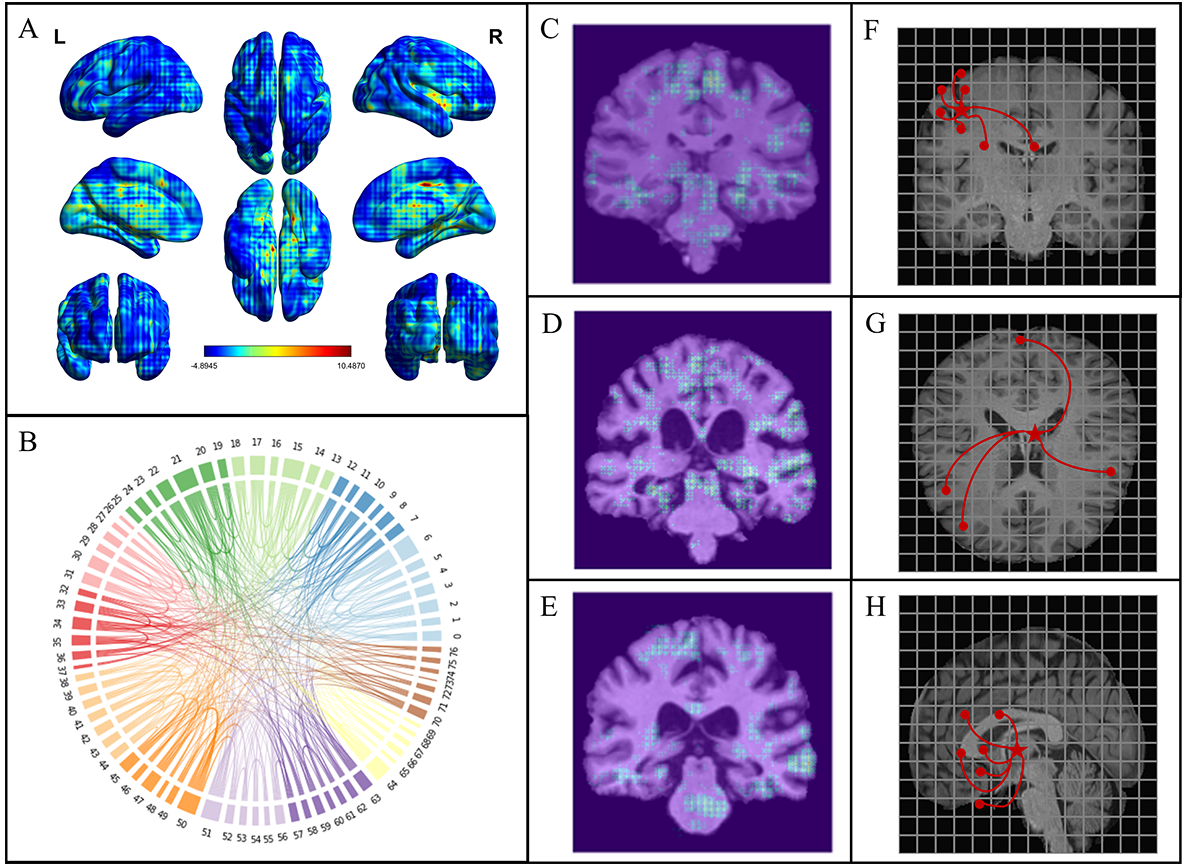
Results for interpretability analysis.

As shown in Figures 4A and 4B, the saliency map indicated that our model exhibited a moderate focus on various dispersed regions within the cortical layer, while notably emphasizing relatively concentrated parenchymal regions. The regions of concern of the model are different in the population identified as CN (Figure 4C) and those with MCI (Figure 4D) and AD (Figure 4E).

In addition, we described the brain as a complex system by extracting edge and node features from the last layer of an isotropic BVGN backbone. In Figure 4B, the top nodes with the highest degree centrality in the final layer of the isotropic BVGN are depicted, showcasing both these central nodes and their immediate neighboring nodes. This visualization provides insights into the most influential regions within the brain’s voxel network as determined by the BVGN’s analysis. Specifically, key cortical areas involved the frontal lobe and temporo-parieto-occipital regions, and parenchymal regions included the corpus callosum, cingulate gyrus, and parahippocampal gyrus, covering the striatum, thalamus, and adjacent hippocampus. In Figures 4F-4H, we leveraged a distinctive visualization approach inherent to the vision GNN backbone to illustrate the spatiotemporal dynamics of interregional aging patterns. The red star marks a specific voxel block, and the most relevant regions are delineated through red line connections.

### Comparison of Brain Age Gap Among Different Cognitive State Groups

**Figure 5:**
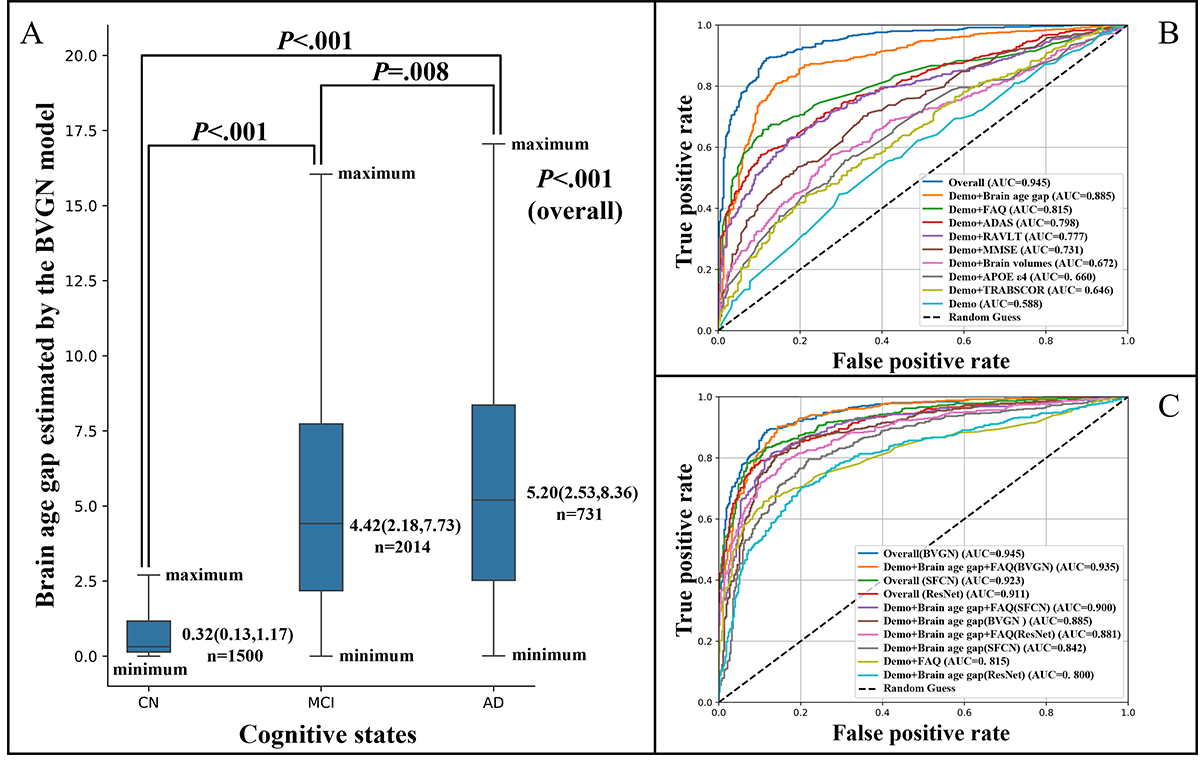
Results or group comparisons and receiver operating characteristic curve of multiple logistic regressions. ADAS: Alzheimer’s Disease Assessment Scale; APOE4: apolipoprotein E 4 alleles; AUC: area under the receiver operating characteristic curve; BVGN: brain vision graph neural network; CN: cognitively normal; FAQ: Functional Activities Questionnaire; MCI: mild cognitive impairment; MMSE: Mini-Mental State Examination; RAVLT: Rey Auditory Verbal Learning Test; ResNet: residual neural network; SFCN: simple fully convolutional network; TRABSCOR: trail making test part B, time.

The median of brain age gap was 0.32 (IQR 0.13-1.17) among 1500 CN samples, 4.42 (IQR 2.18-7.73) across 2014 samples with MCI, and 5.20 (IQR 2.53-8.36) in 731 samples with AD (Figure 5A). A statistically significant difference in brain age gap was observed between AD and MCI (*P*=.008), with MCI demonstrating a significantly higher gap than CN (*P*<.001), indicating the potential of our model to better distinguish between individuals classified as CN and those with MCI. The results adjusted for age and gender are provided in Table S3 in Multimedia Appendix 1, which were similar to the results mentioned earlier.

### Multiple Logistic Regression Models

#### Brain Age Gap Compared to Other Cognition-Related Variables

**Figure 6:**
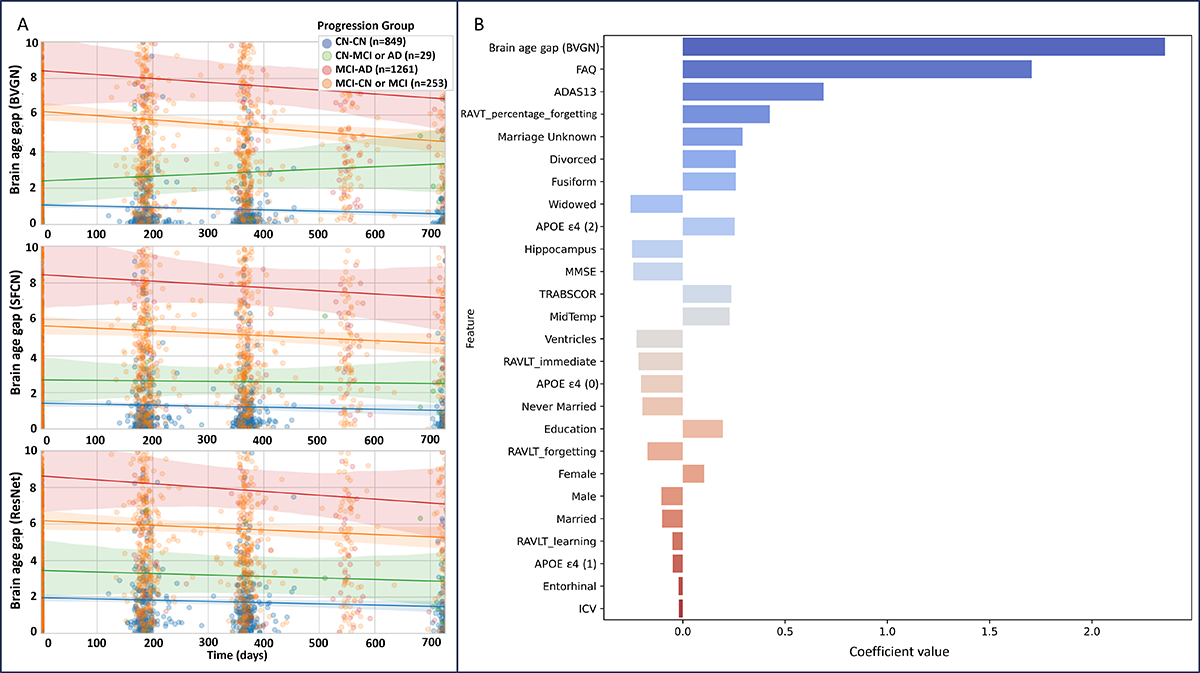
(A) Visualization of longitudinal analysis for brain age gap from various models by progression groups over 2 years and (B) coefficients of various variables in the brain vision graph neural network (BVGN) comprehensive logistics regression model discriminating between cognitively normal (CN) and mild cognitive impairment (MCI). ADAS: Alzheimer's Disease Assessment Scale; APOE ε4: apolipoprotein E 4 alleles; AUC: area under the receiver operating characteristic curve; FAQ: Functional Activities Questionnaire; ICV: intracranial volume; MMSE: Mini-Mental State Examination; RAVLT: Rey Auditory Verbal Learning Test; ResNet: residual neural network; SFCN: simple fully convolutional network; TRABSCOR: trail making test part B, time.

Detailed metrics of various logistic regression analyses aiming to compare brain age gap and other cognition-related variables are provided in Table 4, while multiple receiver operating characteristic curves and corresponding AUC scores are illustrated in Figure 5B. Initially, the benchmark model, including only demographic features, yielded an AUC of 0.588. Subsequent incorporation of additional variables, such as FAQ [47], Alzheimer’s Disease Assessment Scale-Cognitive Subscale13-item version [48], Rey Auditory Verbal Learning Test [49], Mini-Mental State Examination [50], trail making test part B, time [51], brain volume features, APOE4 carriage, and brain age gap led to improved AUC scores, reaching 0.815, 0.798, 0.777, 0.731, 0.646, 0.672, 0.660, and 0.885, respectively. Notably, the comprehensive model encompassing all variables achieved an AUC of 0.945, with the brain age gap exhibiting the highest coefficient (Figure 6B). These results underscore the robust and superior discriminative ability of the brain age gap in distinguishing between CN and MCI samples.

**Table 4:** Performance of the logistics regression models in classifying cognitive states: cognitively normal and mild cognitive impairment (brain age gap versus conventional cognitive variables).

Input features	Accuracy	Precision	Recall	*F*_1_-score	AUC^a^
Overall	0.882	0.915	0.875	0.895	0.945
Demographic+BVGN_BAG^b^	0.820	0.890	0.784	0.833	0.885
Demographic+SFCN_BAG^c^	0.780	0.839	0.764	0.800	0.842
Demographic+ResNet_BAG^d^	0.743	0.823	0.702	0.758	0.800
Demographic+FAQ^e^	0.758	0.880	0.669	0.760	0.815
Demographic+ADAS^f^	0.721	0.774	0.726	0.749	0.798
Demographic+RAVLT^g^	0.714	0.759	0.736	0.747	0.777
Demographic+MMSE^h^	0.678	0.723	0.710	0.717	0.731
Demographic+brain volumes	0.617	0.646	0.734	0.687	0.672
Demographic+APOE4^i^	0.631	0.645	0.794	0.712	0.660
Demographic+TRABSCOR^j^	0.611	0.644	0.720	0.680	0.646
Demographic	0.577	0.583	0.919	0.713	0.588

^a^AUC: area under the receiver operating characteristic curve.

^b^BVGN_BAG: brain age gap estimated by the brain vision graph neural network model.

^c^SFCN_BAG: brain age gap estimated by the simple fully convolutional network model.

^d^ResNet_BAG: brain age gap estimated by the residual neural network model.

^e^FAQ: Functional Activities Questionnaire.

^f^ADAS: Alzheimer’s Disease Assessment Scale.

^g^RAVLT: Rey Auditory Verbal Learning Test.

^h^MMSE: Mini-Mental State Examination.

^i^APOE4: apolipoprotein E ε4 alleles.

^j^TRABSCOR: Trail Making Test Part B Time.

#### Combined Performance of Brain Age Gap With FAQ

In the comprehensive logistics regression model of BVGN, FAQ demonstrated the second-largest coefficient following brain age gap (Figure 6B). When comparing the comprehensive models that included all selective variables with another model incorporating demographic variables, brain age gap and FAQ, minimal declines in AUCs were observed—BVGN decreased by 0.011 (from 0.945 to 0.935), SFCN by 0.024 (from 0.923 to 0.900), and ResNet by 0.031 (from 0.911 to 0.881; Table 5; Figure 5C). Notably, BVGN exhibited the smallest AUC reduction when all variables except FAQ were missing, outperforming other frameworks. In addition, with only demographic variables, brain age gap, and FAQ, BVGN could provide a higher AUC (0.935) compared to other frameworks, even when all selective features were incorporated (SFCN: AUC=0.923; ResNet: AUC=0.911). This underscored the combined efficiency of FAQ and brain age gap, as well as the robustness of BVGN in scenarios with missing variables.

**Table 5:** Performance of logistics regression models: brain age gaps evaluated by models with different precisions (mean absolute errors) and combined utility of brain age gap with Functional Activity Questionnaire (FAQ).

Input features and model			Accuracy		Precision		Recall		*F*_1_-score		AUC^a^
**Overall**											
	BVGN^b^	0.882		0.915		0.875		0.895		0.945	
	SFCN^c^	0.851		0.889		0.845		0.867		0.923	
	ResNet^d^	0.830		0.877		0.819		0.847		0.911	
**Demographic+brain age gap+FAQ**											
	BVGN	0.856		0.907		0.833		0.869		0.935	
	SFCN	0.835		0.895		0.808		0.849		0.900	
	ResNet	0.807		0.873		0.776		0.821		0.881	
**Demographic+brain age gap**											
	BVGN	0.820		0.890		0.784		0.833		0.885	
	SFCN	0.780		0.839		0.764		0.800		0.842	
	ResNet	0.743		0.823		0.702		0.758		0.800	

^a^AUC: area under the receiver operating characteristic curve.

^b^BVGN: brain vision graph neural network.

^c^SFCN: simple fully convolutional network.

^d^ResNet: residual neural network.

#### Potential Clinical Value of Lower MAEs in Brain Age Gap Estimation

In assessing the potential clinical utility of models with lower MAEs, our BVGN model consistently outperformed SFCN and ResNet models (Table 5), both in the simplified model comprising only demographic features and brain age gap and in the comprehensive model incorporating all variables. Notably, brain age gap estimated by BVGN demonstrated the highest AUCs (Figure 5C). Specifically, in the simplified model, BVGN achieved AUCs of 0.885, while SFCN and ResNet yielded AUCs of 0.842 and 0.800, respectively. Similarly, in the comprehensive model, BVGN achieved AUCs of 0.945, surpassing SFCN and ResNet with AUCs of 0.923 and 0.911, respectively. These findings were consistent with the ranking of MAEs obtained from these 3 frameworks, indicating that models with lower MAEs might have the potential to enhance the model’s discriminatory ability.

### Longitudinal Value of BVGN

#### Brain Age Gap Trajectories in 2 Years

The number of MRI scans for CN-CN, CN-MCI or CN-AD, MCI-CN or MCI-MCI, and MCI-AD were 849, 29, 1261, and 253, respectively.

As illustrated in Figure 6A, we observed consistent ordering of brain age gaps across progression groups in all the frameworks over the 2-year period (MCI-AD>MCI-CN or MCI-MCI>CN-MCI or CN-AD>CN-CN). Moreover, brain age gaps tended to decrease over the 2-year follow-up period in each progression group in both the SFCN and ResNet models, as well as in all groups in the BVGN model except the CN-MCI or CN-AD group. However, in the BVGN model, an interesting increase in the brain age gap was observed in the CN-MCI or CN-AD group.

#### Risk Evaluation by Brain Age Gap

Among the 281 individuals classified as CN at the baseline, 12 (4.3%) declined to MCI, while 269 (95.7%) remained in the CN state. Among the 468 participants with MCI at the baseline, 74 (15.8%) declined to AD while 394 (84.2%) remained in the MCI state or improved to the CN state.

Both univariable Cox regression models and demographic-adjusted as well as fully adjusted multivariable models demonstrated that the brain age gap was a significant risk factor for cognitive decline in both the CN and MCI groups (Table S4 in Multimedia Appendix 1). In the univariable model, the HR for brain age gap was 1.55 (95% CI 1.13-2.13) in the CN group and 1.29 (95% CI 1.09-1.51) in the MCI group. For the CN group, the HR for the brain age gap was 1.47 (95% CI 1.06-2.05) in the demographic-adjusted model and increased to 2.13 (95% CI 1.01-4.47) in the fully adjusted model. In the MCI group, the HR was 1.30 (95% CI 1.10-1.53) for the demographic-adjusted model and 1.24 (95% CI 1.03-1.48) for the fully adjusted model.

**Figure 7:**
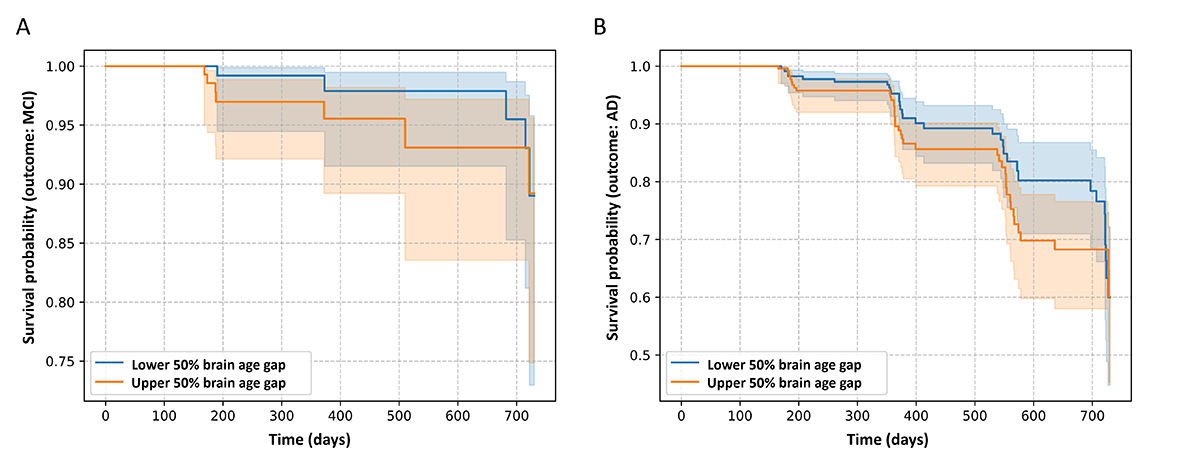
Visualization of Kaplan-Meier curves stratified by the upper and lower 50% levels of brain age gap in both (A) cognitively normal and (B) mild cognitive impairment (MCI) groups. AD; Alzheimer disease.

The Kaplan-Meier curves indicated that in both CN (Figure 7A) and MCI (Figure 7B) groups, the survival curve for the lower 50% of the brain age gap group was higher than that for the upper 50% group. This finding aligned with the HRs, suggesting that a higher level of brain age gap was associated with an increased risk of cognitive decline.

## Discussion

### Principal Findings

In summary, this study proposed a novel framework, BVGN, for brain age estimation. This model not only introduced an innovative modeling approach but also achieved lower MAEs (2.39 years) compared to current state-of-the-art frameworks for brain age gap estimation. It also demonstrated a robust performance on a large-scale external dataset, maintaining strong generalizability (MAE=2.49 years; *r*=0.92; Figure S1 in Multimedia Appendix 1). In addition, BVGN offers an interpretable analysis approach not found in other DL models, which reveals patterns of coaging between brain regions by visualizing the locations of neighboring nodes associated with high-importance nodes. In our study, BVGN provided interpretable attention regions corroborated by medical evidence. Our validation experiments demonstrated its robust performance in MCI diagnosis, its capacity as a risk biomarker for cognitive decline, and its potential to aid in screening and clinical applications.

BVGN integrates deformable convolutional kernels and a dynamic graph for brain age gap estimation. To the best of our knowledge, this work represents the first successful application of vision GNN to T1-weighted MRI data without requiring additional brain segmentation, achieving competitive performance. Unlike 3D CNNs, which process brain images as regular voxel grids and struggle with the inherently irregular morphological characteristics of cerebral regions, often resulting in redundant computations and information loss, BVGN leverages graph-structured representations to preserve complex anatomical features. The integration of deformable convolutional kernels enables the model to focus on specific pathological patterns while suppressing extraneous noise, thereby enhancing local feature modeling [34]. Furthermore, the topological properties of graph structures facilitate multiregional feature aggregation across distant brain regions [38,39], a capability unattainable by grid-based CNNs. To strengthen age prediction accuracy, we transformed age from a discrete numerical variable into a smoothed probability distribution, preserving its continuity and capturing interval similarity [40,41,49,50]. Comparative evaluations demonstrate superior performance of BVGN over existing CNN-based models such as BrainAgeNeXt, achieving an MAE of 2.39 versus 2.78 years, and maintaining robust generalization capabilities (MAE=2.49 years; *r*=0.92) on the UKB dataset after retraining. Structure-functional coupling has garnered increasing attention in neuroscience, with graph-based modeling serving as a critical analytical framework [52]. For instance, structural connectivity derived from diffusion-weighted imaging via white matter fiber tractography and functional connectivity computed from functional MRI are commonly represented as adjacency matrices in graph structures. The framework, being graph-based in design, is inherently more scalable and adaptable to the aforementioned MRI modalities compared to other DL approaches, providing an interface with modal-type embedding for early feature fusion [40]. This capability not only enhances multimodal integration but also helps mitigate the semantic gap across different MRI modalities [53].

We found substantial evidence validating the BVGN’s focused brain regions associated with cognitive decline, which provided strong interpretability for our model. The frontal lobes exhibited particularly prominent changes within an annual 0.87% reduction in overall cortical volume [54], while the temporo-parieto-occipital junction was intricately involved in high-level human neurological functions, such as language, memory, calculation, and writing [55]. Shape characteristics in the corpus callosum were demonstrated to have potential for distinguishing cognitive deterioration [56,57], and the cingulate gyrus was implicated in the onset of neurodegenerative and psychiatric disorders [58]. In addition, the parahippocampal gyrus integrates signals from the limbic and neocortex, predicting later memory [59], and the fractional anisotropy alterations in the parahippocampal white matter have been observed across AD stages [60,61].

In recent years, despite the substantial academic focus on the brain age gap, questions have persisted regarding its reliability as a clinical tool compared to cognitive assessments for diagnosing cognitive decline. After multiple experiments that incorporated various kinds of input variables to distinguish CN and MCI, our findings revealed that except for the trail making test part B, time (AUC=0.646), cognitive assessments exhibited AUCs from 0.731 to 0.815, which surpassed both the AOPE4 carriage (AUC=0.660) and brain volume characteristics (AUC=0.672), and were inferior to brain age gap (AUC=0.885). While cognitive assessments provided affordability, the subjective bias among clinical practitioners might impact diagnosis [62], with limitations in sensitivity observed in certain cases, such as mild levels of impairment [32], subjective deficits, and healthy cognitive aging [63]. According to our findings, our model could offer alternative and more objective evaluations for clinical settings, potentially reducing physicians’ workload and enhancing convenience.

The FAQ is a 10-item collateral-report scale with 4-point ordinal responses per item, totaling 0 to 30 points, with higher scores indicating greater functional impairment [64]. This makes it highly accessible in clinical settings and convenient for clinical practitioners. Scientific publications have demonstrated its good internal consistency and high discriminative validity for differentiating between CN and MCI, as well as between MCI and very mild AD [65,66]. Its high predictive validity in detecting individuals at risk of progression from MCI to AD and from CN to MCI has also been reported [47]. In our study, we observed minimal reductions in AUCs when comparing models that incorporated all selective variables and models with only demographic variables, brain age gap, and FAQ. Our selected demographic variables, education years, gender, and marriage status, were deliberately straightforward. This simplicity would enhance the feasibility of our proposed model for clinical applications, especially in cases of uncertain MCI diagnosis. By integrating basic demographics, FAQ responses, and T1-weighted MRI scans, our BVGN model could serve as a reliable assistant tool for clinicians in MCI diagnosis.

In our comprehensive model that integrated brain age gap, cognitive assessments, and other relevant variables, we observed the highest AUC of 0.945 in classification tasks. The integration of variables from diverse perspectives might contribute to improving performance. In addition, we observed brain age gap possessed the highest variable coefficient, with each scalarized unit increase associated with a 10.6 times higher risk of MCI. This further supported the brain age gap’s capacity in MCI diagnosis.

Furthermore, our analysis revealed a relationship between model performance, as indicated by lower MAEs, and superior performance in discriminating between CN and MCI. Specifically, BVGN, SFCN, and ResNet achieved MAEs of 2.39, 2.41, and 2.65 years, respectively. Their corresponding AUCs in the simplified and comprehensive logistics models for classifying CN and MCI were 0.885, 0.842, and 0.800 and 0.945, 0.923, and 0.911. This finding underscored the importance of striving for minimal MAE in model development for enhanced predictive accuracy in subsequent cognitive decline tasks.

During longitudinal analysis, we observed an increase in the brain age gap derived from BVGN in CN-MCI or CN-AD progression group within 2 years after baseline. This increase was not observed in either SFCN or ResNet models. This group of participants classified as “CN” might be some individuals at high risk who have already experienced early pathological changes related to dementia yet remain classified as “CN” due to the absence of noticeable cognitive impairments. This finding supported BVGN as a sensitive marker for identifying participants at high risk who, despite being classified as “CN,” were likely to progress to MCI in a few years. Existing scientific research suggests that MCI can be reversible to a certain extent, whereas AD, which is associated with significant cognitive degeneration, is challenging to reverse once it has progressed [67-69]. Therefore, early identification of these individuals at high risk for MCI is of great significance for better patient prognosis.

Multiple Cox proportional hazard models and Kaplan-Meier curves revealed a clear association between a higher brain age gap and an increased risk of cognitive decline. Specifically, each 1-unit increase in the brain age gap was associated with a 55% higher risk of cognitive decline in the CN group and a 29% increased risk in the MCI group. This finding underscored the brain age gap derived from BVGN as a sensitive risk biomarker for cognitive decline. Notably, the brain age gap appeared more sensitive in the CN groups. This observation aligned with existing research, which indicated the ability of the brain age gap to detect subtle preclinical or early neurodegenerative changes in populations classified as CN [70,71].

In clinical settings, the BVGN-estimated brain age gap serves as a tool for detecting cognitive decline and assessing the efficacy of innovative therapeutic interventions. For example, in the United States, 2 antiamyloid monoclonal antibodies, lecanemab (brand name Leqembi) and aducanumab (brand name Aduhelm) [72,73], have been approved for the treatment of AD. Despite the potential therapeutic benefits, these interventions are not devoid of inherent risks. Patients require vigilant monitoring for potential side effects, such as amyloid-related imaging abnormalities and infusion reactions, particularly at the onset of treatment. Currently, imaging provides limited insight into a patient’s therapeutic response. However, BVGN could theoretically offer a novel approach by capturing changes in brain age pre- and posttreatment with monoclonal antibodies. This could provide an objective measure of a patient’s response to the medication, enhancing our ability to assess the efficacy of these treatments.

### Limitations and Future Directions

The limitation of BVGN lies in its inability to evaluate the performance and interpretability analysis results on larger datasets. In addition, the use of 3D deformable convolutional kernels in BVGN leads to increased computational costs during the training process when compared to other DL models. Our proposed DL-based brain aging framework is specifically designed for the early screening and prediction of neurodegenerative diseases. Epidemiological and neuroimaging studies indicate that structural alterations linked to neurodegenerative processes begin to manifest or accelerate after the age of 50 years [74]. To better capture brain age deviations closely associated with these pathological conditions, we selected individuals aged >45 years as our study population. This age threshold ensures the inclusion of individuals at the critical transition phase where neurodegeneration-related biomarkers become detectable. However, extending this framework to the entire life span remains essential to comprehensively understand age-related neurodegenerative processes and identify potential early intervention strategies across all life stages.

Despite its limitations, BVGN remains a valuable tool in the study of brain aging, as it surpasses cognitive assessments, brain volume features, and susceptibility genes in classifying participants as CN and those with MCI, and has been demonstrated to be a risk factor for cognitive decline. This underscores the effectiveness of the brain age gap, estimated through individual MRI using BVGN, as an impactful neuroimaging biomarker. Furthermore, BVGN introduces graph theory analysis as a novel component of interpretability for the first time in brain age gap estimation. In the future, we will leverage the advantages of GNNs in brain analysis, and integrate them with other modalities of medical imaging to further enhance our framework and provide a more robust interpretability analysis based on graph theory. Future work will focus on 3 key directions to enhance the clinical utility and scientific depth of our framework. First, we will explore diverse graph convolutional feature aggregation mechanisms to optimize computational efficiency, enabling broader deployment in real-world clinical environments. Second, we aim to integrate multimodal MRI data by converting different imaging sequences (eg, T1-weighted, T2-weighted, and fluid attenuated inversion recovery) into graph-based representations within the BVGN framework. This multimodal integration will not only enhance the accuracy of brain aging quantification but also enable systematic investigation of cross-modal interactions underlying neurodegenerative processes. Finally, we plan to extend our framework across the entire life span to uncover age-specific aging patterns and their associations with disease progression.

### Conclusions

This study proposed a novel DL framework, BVGN, for accurate brain age estimation. Our BVGN model used deformable convolutional kernel to capture brain complex morphology and graph theory to model brain topology, which was applicable to the biological nature of brain MRI. BVGN achieved a superior MAE of 2.39 years in the same testing set compared to other methods, with its attention region aligning with medical evidence of cognitive decline. The robust capacity of BVGN-derived brain age gap was validated through both cross-sectional and longitudinal analysis, which outperformed all conventional tools in aiding the diagnosis of MCI and was sensitive for identifying individuals at high risk for future MCI. The BVGN model can precisely evaluate brain aging and predict cognitive decline, offering substantial potential for improving early identification of neurodegenerative disorders and enhancing clinical applications.

## Appendix

Multimedia Appendix 1 Supplementary tables, figures, and detailed brain vision graph neural network (BVGN) architecture: exhaustive demographics for BVGN development and generalizability validation, Dunn-corrected intergroup comparisons, Cox regression results for brain-age-gap risk, and scatter plot performance across external cohorts.

## Abbreviations

AD: Alzheimer disease
ADNI: Alzheimer’s Disease Neuroimaging Initiative
AUC: area under the receiver operating characteristic curve
BVGN: brain vision graph neural network
CN: cognitively normal
CNN: convolutional neural network
DL: deep learning
FAQ: Functional Activities Questionnaire
GNN: graph neural network
HR: hazard ratio
MAE: mean absolute error
MCI: mild cognitive impairment
MRI: magnetic resonance imaging
ResNet: residual neural network
SFCN: simple fully convolutional network
UKB: UK Biobank
