# Supplementary material for: Development and Validation of a Brain Aging Biomarker in Middle-Aged and Older Adults: Deep Learning Approach
Source: JMIR Aging. 2025 Aug 1;8:e73004. doi: 10.2196/73004 (PMC12357125; doi:10.2196/73004)
Supplement: Multimedia Appendix 1 [file aging_v8i1e73004_app1.docx]

**Multimedia Appendix 1**

**Text S1: Detailed Caption for Figure 2**

The BVGN consists of three layers: (1) The Discretization layer is utilized to partition the 3D standard brain MRI in MNI152 space, obtained through center-cropping, into multiple cubes. It incorporates a learnable parameter matrix that represents positional information and tokenized gender feature vectors. (2) The stacked graph neural network layers begin with a 3-axis average pooling layer for dimensionality reduction, followed by a message-passing based graph neural network to extract features that encompass topological information. (3) The probability distribution prediction layer employs 1×1 convolutional kernel to reduce the channel dimensions from the preceding layer, aligning them with the number of age intervals, and subsequently generates an output representing the probability distribution of ages. *Figure 2A*: A preprocessing pipeline was implemented, which involved converting gender into one-hot encoding and transforming raw MRI data into brain tissue located in a standard space. *Figure 2B*: Discretization layer, the voxel space of brain is divided by the discretization layer, which employs a combination of multiple convolution modules and deformable convolution modules. Subsequently, positional features and gender features are integrated into cubes. *Figure 2C*: Orthogonal average pooling module (Coronal axis, Sagittal axis, Transverse axis, the 3D feature cube is transformed into a 2D feature map. *Figure 2D*: Stacked Graph Neural Network modules. *Figure 2E*: During training, the image on the right illustrates that the supervised signal of backpropagation is computed by evaluating the Kullback-Leibler divergence between estimated brain age and chronological age; whereas the image on the left depicts the model’s performance.

**Table S1: Demographic Characteristics of Dataset for Testing BVGN’s Generalizability**

| Variables | Total dataset (n=34,352) | Training & Validating dataset (n=26,107) | Testing dataset (n=8,245) | Statistic | *P* value |
| --- | --- | --- | --- | --- | --- |
| Age (year), mean (SD) | 64.35 (7.72) | 64.34 (7.69) | 64.40 (7.80) | -0.62 | 0.53 |
| Gender, n (%) |  |  |  | 1.08 | 0.30 |
| Male | 16,335 (47.6) | 12,456 (47.7) | 3,879 (47.1) |  |  |
| Female | 18,017 (52.4) | 13,651 (52.3) | 4,366 (52.9) |  |  |

**Table S2: Demographic Characteristics for Dataset of BVGN Development**

| Variables | Total dataset (n=1920) | Training & Validating dataset (n=1536) | Testing dataset (n=384) | Statistic | *P* value |
| --- | --- | --- | --- | --- | --- |
| Age (year), mean (SD) | 75.70 (6.37) | 75.65 (6.31) | 75.89 (6.60) | -0.66 | 0.51 |
| Gender, n (%) |  |  |  | 1.50 | 0.22 |
| Male | 891 (46.4) | 724 (47.1) | 167 (43.5) |  |  |
| Female | 1029 (53.6) | 812 (52.9) | 217 (56.5) |  |  |

**Table S3: Dunn’s Test^a^ Results (Corrected Age and Gender) for Inter-group Comparisons**

|  | CN | MCI | AD |
| --- | --- | --- | --- |
| CN | 5.000000e-01 | 7.171021e-204 | 1.103137e-199 |
| MCI | 7.171021e-204 | 5.000000e-01 | 5.855107e-14 |
| AD | 1.103137e-199 | 5.855107e-14 | 5.000000e-01 |

^a^Dunn’s Test: Gender was encoded as “1” for “Female” and “0” for “Male”. Cognitive state was encoded as “0” for “CN”, “1” for “MCI”, “2” for “Dementia”. Method: Least Square; Model: OLS; Adjusted R-squared: 0.301; AIC: 2.507e+04; BIC: 2.501e+04.

**Table S4: Risk of Brain Age Gap on Cognitive Decline in CN and MCI Populations (Cox Regression Results)**

| Group | Outcome | Model | HR | HR (95%CI) | *P* value |
| --- | --- | --- | --- | --- | --- |
| CN  (n=281) | Decline to MCI  (12 of 281) | Univariate Model^a^ | 1.55 | (1.13,2.13) | 0.006 |
|  |  | Multivariate Model 1^b^ | 1.47 | (1.06,2.05) | 0.020 |
|  |  | Multivariate Model 2^c^ | 2.13 | (1.01,4.47) | 0.046 |
| MCI  (n=468) | Decline to AD  (74 of 468) | Univariate Model^a^ | 1.29 | (1.09,1.51) | 0.002 |
|  |  | Multivariate Model 1^b^ | 1.30 | (1.10,1.53) | 0.002 |
|  |  | Multivariate Model 2^c^ | 1.24 | (1.03,1.48) | 0.021 |

^a^Univariate Model: no covariates adjusted

^b^Multivariate Model 1: adjusted for demographic variables (gender, education and marriage states)

^c^Multivariate Model 2: adjusted for all selected variables


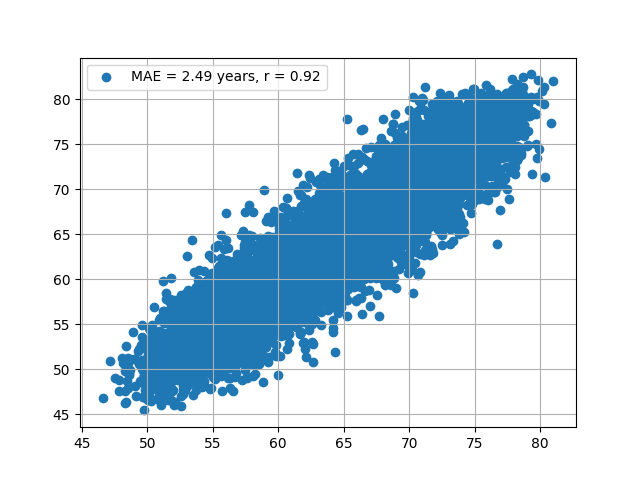


**Figure S1: Performance of BVGN’s Generalizability.**
